# Lidocaine injection of pericranial myofascial trigger points in the treatment of frequent episodic tension-type headache

**DOI:** 10.1186/1129-2377-14-44

**Published:** 2013-05-22

**Authors:** Ömer Karadaş, Hakan L Gül, Levent E İnan

**Affiliations:** 1 Neurology Service, Erzincan Military Hospital, Erzincan, Turkey; 2 Department of Neurology, Kartal Education and Research Hospital, Istanbul, Turkey; 3 Department of Neurology, Ankara Education and Research Hospital, Ankara, Turkey

**Keywords:** Tension-type headache, Lidocaine, Trigger point

## Abstract

**Background:**

The present study aimed to evaluate the efficacy of local lidocaine injections into the myofascial trigger points (TPs) located at the pericranial muscles in patients with episodic tension-type headache (ETTH).

**Methods:**

The study included 108 patients with frequent ETTH that were randomized into 4 groups. One injection of saline (NaCl 0.9%) was administered to group 1 (n = 27), 1 injection of lidocaine (0.5%) was administered to group 2 (n = 27), group 3 (n = 27) received 5 injections of saline (NaCl 0.9%), and group 4 (n = 27) received 5 injections of lidocaine (0.5%); on alternate days 2 mL for each muscle was injected into the frontal, temporal, masseter, sternocleidomastoid, semispinalis capitis, trapezius and splenius capitis muscles bilaterally. The frequency of painful days per month (FPD) and the patients’ visual analogue scales (VAS) were evaluated before treatment, and 2, 4 and 6 months after treatment.

**Results:**

Mean age of the patients was 36.28 ± 9.41 years (range: 18–54 years). FPD scores improved significantly in group 2, 3 and 4 at 2 months posttreatment compared to pre- treatment (all P < 0.05), and also VAS scores improved significantly in group 2 and 4 at 2 months posttreatment (P < 0.05) but this improvement insisted at the 6 month only in group 4. Group 2 had better VAS and FPD than group 1 only at 2. and 4. months after treatment (for VAS P < 0.0121, P = 0.0232; for FPD P = 0.0003, P = 0.0004, respectively). Group 4 had better scores than group 3 at the 2., 4. and 6. months after treatment in both parameters (all P < 0.05). Group 2 had better scores than group 1 in FPD at the 2. and 4. months posttreatment (P = 0.0003, P = 0.0004, respectively), but not at the 6. month.

**Conclusion:**

Local lidocaine injections into the myofascial TPs located in the pericranial muscles could be considered as an effective alternative treatment for ETTH.

## Background

Headache is one of the most common symptoms in the general population. It is important to emphasize that headache can be a symptom of serious pathology and can be due to many etiologies [1]. According to population-based studies, the annual prevalence rates are 38.3% for episodic tension-type headache (ETTH) and 2.2% for chronic tension-type headache (CTTH) [2]. The prevalence of tension-type headache (TTH) is highest between the 2nd and 5th decades of life, peaking between 30 and 40 years of age [3,4].

It remains unclear if TTH is caused by peripheral mechanisms or by the central nervous system (CNS) [5,6]. One reported hypothesis is that altered pain modulation due to nociceptive stimuli arising from pericranial myofascial tissues may cause sensitization in the trigeminal nucleus, thalamus, or somatosensory cortex [7]. Another study reported a decrease in the gray matter volume in patients with CTTH, suggesting that this condition is the result of central sensitization caused by stimuli arising in pericranial myofascial tissues [8]. Peripheral mechanisms also play a major role in TTH. Prolonged peripheral nociceptive input has been reported to increase sensitization to peripheral pain via sensitization of the central mechanisms, resulting as CTTH [9,10].

Substance P released from muscle afferents and calcitonin gene-related peptide (CGRP) also play a role in myofascial pain [11]. A placebo-controlled study by Jensen and Olsen reported that experimental tooth clenching for 30 minutes triggered TTH in patients with CTTH or ETTH. Their results showed that 69% of the patients developed TTH within 24 hours, versus 17% of the control group. Pain tolerance decreased in patients that developed TTH, but no change was observed in the other participants. Their results also suggested that insufficient activation of the antinociceptive system in the patients with TTH and peripheral mechanisms might play a role in the pathogenesis of TTH by triggering the central mechanisms [12]. Hu et al. [13] reported that neurons in the trigeminal nucleus (located in the brainstem) were sensitized via stimulation of craniofacial muscle afferents.

Treatment of TTH includes both acute and prophylactic treatments. Analgesics are used for the acute treatment of TTH, whereas antidepressants are used for prophylaxis. These drugs’ side effects and drug interactions must be taken into consideration during such treatment [14]. Non-pharmacological pain management alternatives may also be considered; however, current data have not proven that they are efficacious [15].

The aim of the present study was to evaluate the efficacy of local lidocaine injections into the myofascial TPs at the pericranial muscles, to regulate the antinociceptive system in the treatment of frequent ETTH.

## Methods

The study included patients aged between 18 and 65 years that were diagnosed with frequent ETTH according to the criteria published in 2004 by the International Headache Society (IHS) [16], that had normal physical and neurological examination results, and had headache less than 15 days per month during the previous 6 months. All patients were evaluated ragarding the other causes of headache and patients who had headache with a cause except than ETTH were excluded from the study. Our study was approved by Local Ethics Committee and informed consent forms were obtained from all participants.

Exclusion criteria:

•Patients responding to medical treatment

•Patients using more than 15 doses of analgesics in a month

•Patients who received botulinum toxin type A (BoNT-A) therapy

•Pregnant women

•Patients with known allergies against local anesthetics

•Patients with a history of malignancy

•Patients who had a history of cervical and cranial surgery

•Patients who had received non-pharmacological therapy for the last 6 months

•Patients with anemia and bleeding diathesis

•Patients with major psychiatric disorders (major depression etc.)

•Patients who used antipsychotic, antidepressant and antiepileptic drugs within the previous 3 months

•Patients with neuromuscular dysfunction

•Patients with uncontrolled hypertension

•Patients with hypothyroidism or hyperthyridism

Whole blood count, routine biochemistry, and thyroid function tests were performed, and vitamin B-12, folate, and ferritin levels were measured in each patient to exclude possible secondary causes of frequent ETTH. Computed tomography (CT) or magnetic resonance imaging (MRI) were performed when considered to be necessary.

This was a double-blind placebo controlled randomized study. Patients were randomly assigned into four groups. The study medication was prepared by a registered nurse. The study medication was delivered to the injecting physician in identical appearing syringes by a different nurse. Each syringes contained an identical amount of solution. Evaluations and injections were performed by separate physicians. Neither the physician nor the patient knew which treatment to be administered. The evaluator was blind to treatment groups. The study protocol was approved by the Local Ethics Committee and patients had signed informed consent forms. In all, 108 patients were included to the study, following implementation of inclusion and exclusion criterias. Pain severity in patients was evaluated using the visual analogue scale (VAS) and the frequency of painful days per month (FPD) were recorded before the treatment.

### Procedure

Patients were randomly divided into 4 groups. One injection of saline (NaCl 0.9%) was administered to group 1 (n = 27), 1 injection of lidocaine (0.5%) was administered to group 2 (n = 27), group 3 (n = 27) received 5 injections of saline (NaCl 0.9%), and group 4 (n = 27) received 5 injections of lidocaine (0.5%): on alternate days 2 mL for each muscle was injected into the frontal, temporal, masseter, sternocleidomastoid, semispinalis capitis, trapezius and splenius capitis muscles bilaterally. Injections were perfomed into the trigger points of the muscles. We detected pericranial myofascial trigger points by making small circular movements, applying deep finger pressure (finger tipping) on the surface of the muscles with systematic palpation as explained in Travel & Simons manual [17]. All the patients have headache bilaterally, and myofascial trigger points were detected bilaterally in all patients. Lidocaine injections were performed to same muscles (injections were made to myofascial trigger points of the muscles that have dominant areas with pain) and with standard doses. Applications in repetetive injections were made to the same trigger points.

The patients’ VAS and FPD were evaluated before treatment, and 2, 4, and 6 months after treatment. The efficacy of the injections was evaluated 2, 4, and 6 months after treatment by a physician (different than the one that administered the injections) blinded to patients group.

Statistical analysis was performed using SPSS software for Windows, version 15.0. Mann Whitney U test was used for non-parametric unpaired values with a group number equal to 2. For parametric unpaired data with a group number equal to 2, the unpaired t-test was used. In nonparametric unpaired data with more than 2 groups, the Kruskal Wallis test was used. In parametric data with more than 2 groups, the one way analysis of variance (ANOVA) was used.

## Results

The present study included 108 patients (32 male, 76 female). The mean age of the patients was 36.28 ± 9.41 years (range: 18–54 years). Demographic characteristics of the patients are summarized in Table 1. There weren’t any differences in age or gender between the groups (P > 0.05).

**Table 1:** Patient demographics

	**Group 1**	**Group 2**	**Group 3**	**Group 4**
**n (Male/Female)**	8/19	7/20	9/18	8/19
**Age (years)**	36,1 ± 9,7	37,03 ± 8,1	35,96 ± 9,5	36,03 ± 10,6

As observed in Table 2: the mean VAS scores during pretreatment and at the 2., 4. and 6. month in group 1 were found to be 71.5 ± 7.6, 68.0 ± 9.0, 69.8 ± 6.6 and 70.2 ± 6.7, in group 2: 70.4 ± 8.4, 59.8 ± 13.6, 64.1 ± 10.9 and 68.9 ± 7.3, in group 3: 70.7 ± 6.9, 66.1 ± 8.6, 69.3 ± 6.3 and 69.2 ± 6.2 and in group 4: 71.1 ± 10.2, 48.5 ± 13.7, 55.6 ± 11.5 and 58.7 ± 12.7, respectively.

**Table 2:** Comparison of the data of visual analogue scale belonging to all groups before and after treatment

		**G1**	**G2**	**G3**	**G4**	* p value
** VAS before treatment**	N	27	27	27	27	0.9664
	Mean	71.5	70.4	70.7	71.1	
	SD	7.6	8.4	6.9	10.2	
	Minimum	60	55	60	50	
	Maximum	85	85	85	85	
**VAS for 2 months after treatment**	Mean	68.0	59.8	66.1	48.5	< 0.0001
	SD	9.0	13.6	8.6	13.7	
	Minimum	50	35	50	20	
	Maximum	85	80	85	80	
	Comparison	G1 vs G2	G1 vs G3	G2 vs G4	G3 vs G4	
	p value	^a^ 0.0121	^a^ 0.4431	^a^ 0.0037	^a^ <0.0001	
**VAS for 4 months after treatment**	Mean	69.8	64.1	69.3	55.6	< 0.0001
	SD	6.6	10.9	6.3	11.5	
	Minimum	50	40	50	40	
	Maximum	80	80	80	80	
	Comparison	G1 vs G2	G1 vs G3	G2 vs G4	G3 vs G4	
	p value	^a^ 0.0232	^a^ 0.7527	^a^ 0.0075	^a^ <0.0001	
**VAS for 6 months after treatment**	Mean	70.2	68.9	69.6	58.7	< 0.0001
	SD	6.7	7.3	6.2	12.7	
	Minimum	50	55	50	30	
	Maximum	80	85	80	80	
	Comparison	G1 vs G2	G1 vs G3	G2 vs G4	G3 vs G4	
	p value	^a^ 0.4987	^a^ 0.7533	^a^ 0.0007	^a^ 0.0002	

N: Number of patients, SD: Standard deviation, * One-way Analysis of Variance (ANOVA), ^a^ Unpaired t test. VAS: Visual Analogue Scale. If p value obtained by ANOVA is <0.05, p values of between groups (G1,G2,G3 and G4) are compared.

**Figure 1:**
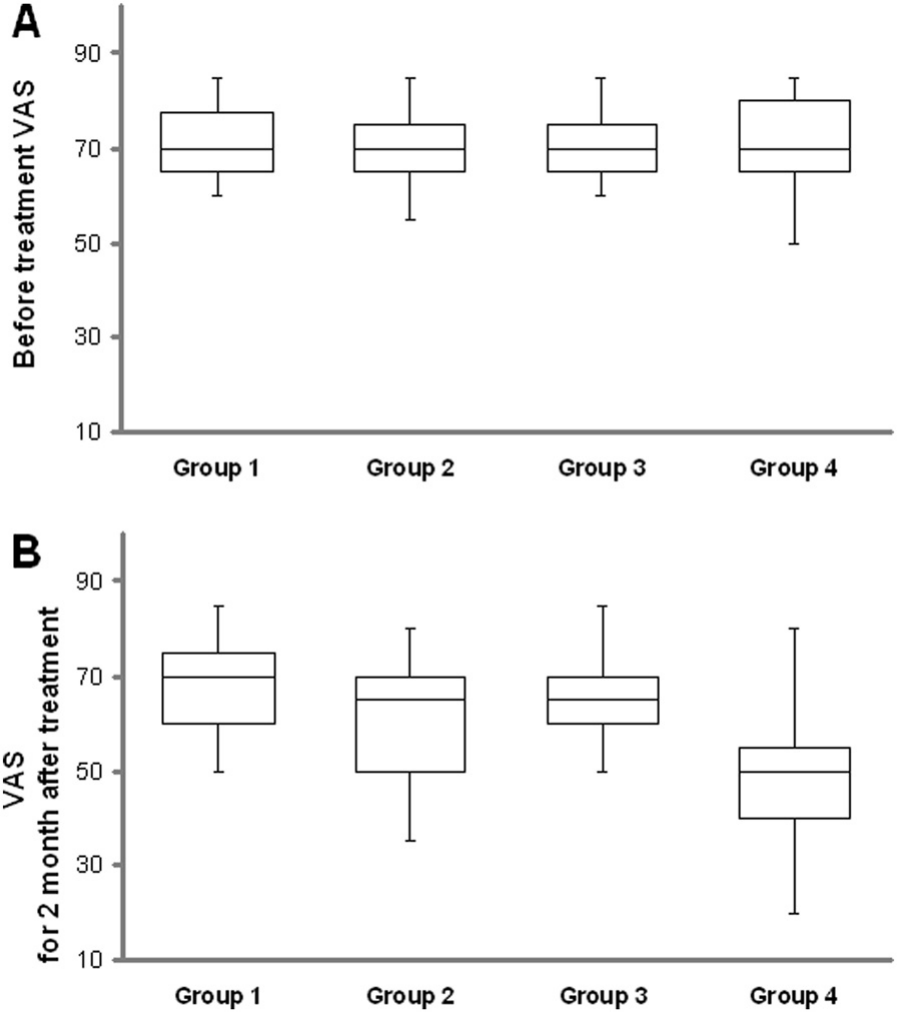
An illustration of all groups, the distribution of values for visual analogue scale (VAS): (A) before treatment and (B) 2 months after treatment.

**Figure 2:**
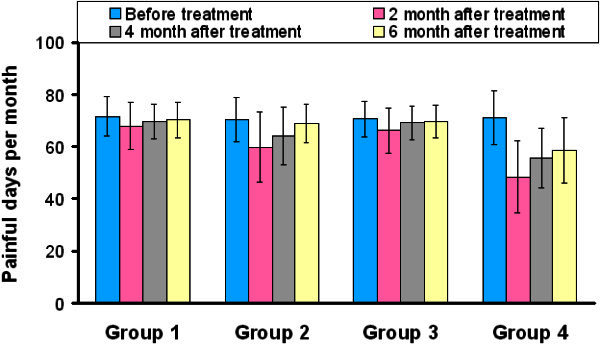
Graph showing the frequency of painful days before and after treatment in all groups.

**Figure 3:**
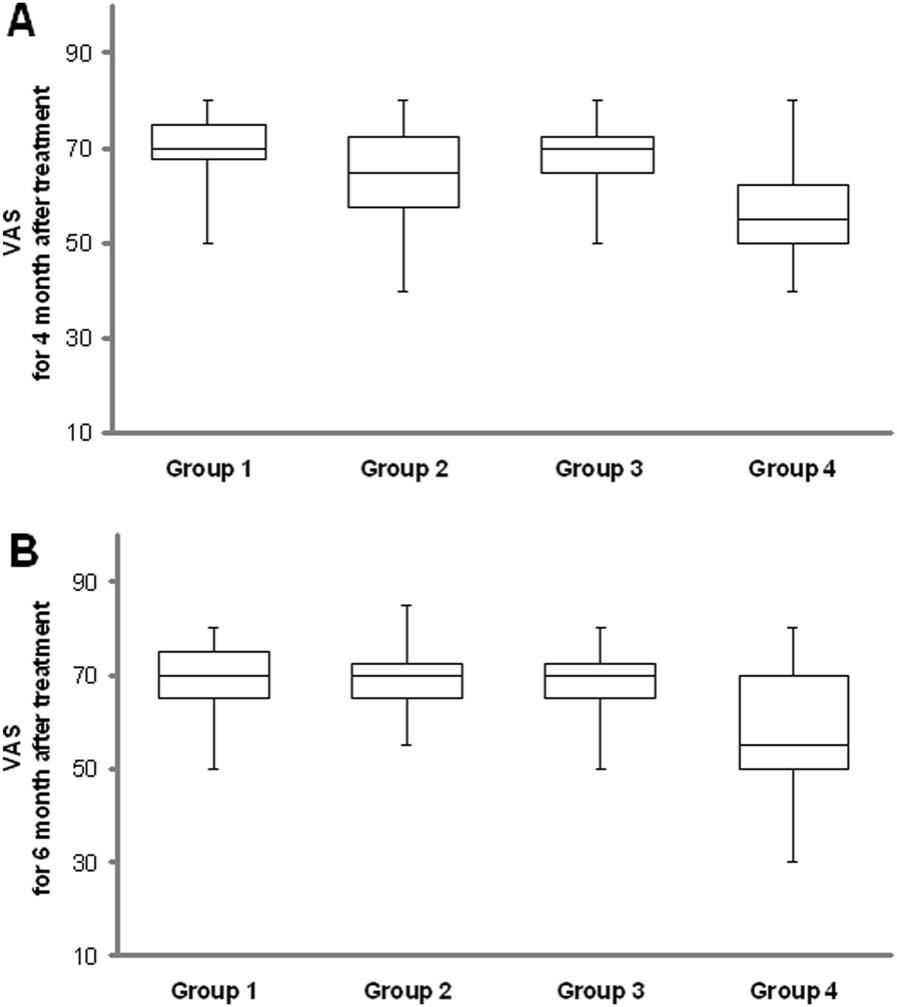
An illustration of all groups, the distribution of values for visual analogue scale (VAS) for (A) 4 month after treatment and (B) 6 months after treatment.

All the groups were similar in terms of VAS and FPD before treatment (all P > 0.05) (Table 2 and Table 3 and Figures 1A and 2). FPD scores improved significantly in group 2, 3 and 4 at the 2. month posttreatment when compared with pretreatment (all P < 0.05), and also VAS scores improved significantly in group 2 and 4 at the 2. month posttreatment (P < 0.05) but this improvement insisted at the 6. month only in group 4 (Table 4 and Figures 1B, 2 and 3). Group 2 had better VAS and FPD scores than group 1 only at the 2. and 4. months after treatment (all P < 0.05). Group 4 had better scores than group 3 at the 2., 4. and 6. months after treatment in both parameters (all P < 0.05) (Figures 1B, 2 and 3).

**Table 3:** Comparison of the frequency of painful days belonging to all groups before and after treatment

		**Group 1**	**Group 2**	**Group 3**	**Group 4**	* p
** FPD before treatment**	N	27	27	27	27	0.9272
	Mean	11,1	10,8	11,0	11,0	
	SD	1,7	1,8	1,6	2,2	
	Minimum	8	7	8	7	
	Maximum	14	14	13	14	
**FPD for 2 months after treatment**	Mean	10,4	8,4	9,9	6,6	<0.0001
	SD	1,2	2,4	1,3	2,2	
	Minimum	9	4	7	2	
	Maximum	12	13	12	10	
	Comparison	G1 vs G2	G1 vs G3	G2 vs G4	G3 vs G4	
	p value	^a^ 0.0003	^a^ 0.1708	^a^ 0.0071	^a^ <0.0001	
**FPD for 4 months after treatment**	Mean	10,9	8,8	10,7	7,6	<0.0001
	SD	1,6	2,3	1,6	1,9	
	Minimum	8	5	8	4	
	Maximum	14	13	14	11	
	Comparison	G1 vs G2	G1 vs G3	G2 vs G4	G3 vs G4	
	p value	^a^ 0.0004	^a^ 0.8007	^a^ 0.0466	^a^ <0.0001	
**FPD for 6 months after treatment**	Mean	11,1	10,3	10,9	7,9	<0.0001
	SD	1,4	2,4	1,5	2,0	
	Minimum	9	6	8	4	
	Maximum	14	15	14	11	
	Comparison	G1 vs G2	G1 vs G3	G2 vs G4	G3 vs G4	
	p value	^a^ 0.1506	^a^ 0.7068	^a^ 0.0003	^a^ <0.0001	

N: Number of patients, SD: Standard deviation, * One-way Analysis of Variance (ANOVA), FPD: Frequency of painful days per month. If p value obtained by ANOVA is <0.05, p values of between groups (G1,G2,G3 and G4) are compared.

**Table 4:** Visual analogue scale and frequency of painful days per month scores before and after treatment in all groups

	**Comparison of the VAS data before and after treatment**							**Comparison of the FPD data before and after treatment**						
	**BT vs 2 month AT**	**BT vs 4 month AT**	**BT vs 6 month AT**	**2 month vs 4 month AT**	**2 month vs 6 month AT**	**4 month vs 6 month AT**	**p Value**	**BT vs 2 month AT**	**BT vs 4 month AT**	**BT vs 6 month AT**	**2 month vs 4 month AT**	**2 month vs 6 month AT**	**4 month vs 6 month AT**	**p Value**
**Group 1**	ª -	* 0.131	<0.05	>0.05	>0.05	>0.05	>0.05	>0.05	* 0.029					
**Group 2**	<0.001	<0.05	>0.05	>0.05	<0.001	>0.05	* <0.001	<0.001	<0.001	>0.05	>0.05	<0.001	<0.01	* <0.001
**Group 3**	<0.01	>0.05	>0.05	>0.05	<0.05	>0.05	* 0.0045	<0.001	>0.05	>0.05	<0.01	<0.001	>0.05	* <0.001
**Group 4**	<0.001	<0.001	<0.001	<0.05	<0.001	>0.05	* <0.001	<0.001	<0.001	<0.001	<0.05	<0.01	>0.05	* <0.001

BT: Before treatment, AT: After treatment, VAS: Visual analogue scale, FPD: frequency of painful days per month, * p Value for Repeated Measures ANOVA (Analysis of Variance) with post test, ª Post tests were not calculated because the p value was greater than 0.05.

**Figure 4:**
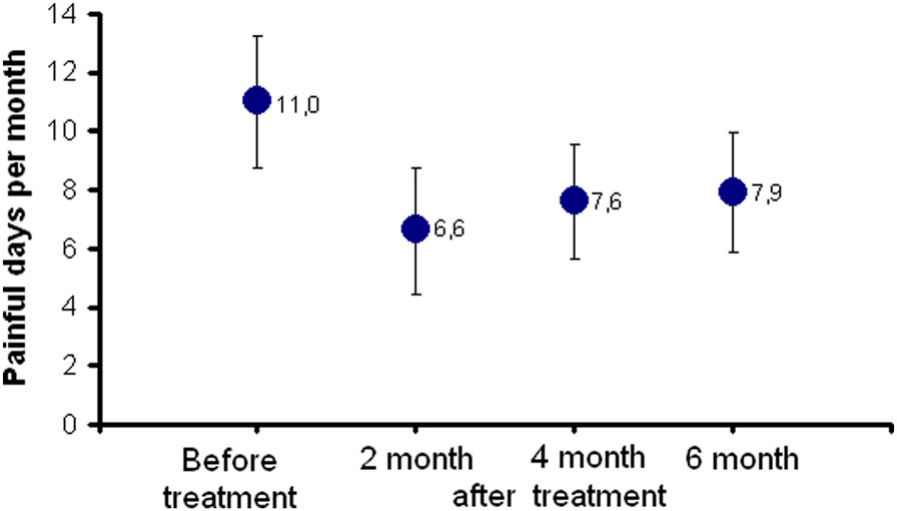
**Comparison of the frequency of painful days before and after treatment belonging to the group received injection of lidocaine hydrochloride 5 times.** A significant decrease in the frequency of painful days after treatment (2, 4 and 6 month) compared to pre-treatment was observed P < 0.001). 2., 4. and 6. Months after the treatment, there was no difference between the frequency of painful days (P > 0.005).

When the FPD in group 4 were compared pre- and posttreatment (2, 4 and 6 mounth), all posttreatment FPD scores compared to pretreatment scores was significantly lower (Figure 4).

No serious side effects leading to the termination of the treatment were observed; other side effects are shown in Table 5.

**Table 5:** The frequency of side effects

**Side Effects**	**Group 1**	**Group 2**	**Group 3**	**Group 4**
Pain at injection area	1	1	1	1
Dizziness	1	0	1	1
Servical muscle spasm	1	0	0	1

## Discussion

The present study demonstrated clinical benefit among lidocain treated frequent ETTH patients. The results showed that 5 lidocaine injections on alternate days significantly reduced both the frequency and severity of pain at the 2., 4. and 6. months post treatment, as compared to the placebo; in addition, patients with frequent ETTH responded better to repeated lidocaine injection treatment.

Most commonly, frequent TTH affects individuals between the ages of 30 and 40 years [3,4]. Mean age of the patients in the present study was 36.7 years. It remains unclear if the primary origin of TTH is peripheral or central; however, it is conceivable that both peripheral and central mechanisms play a role in TTH. In order to re-establish pain control, it seems that it is possible to regulate the central response by controlling prolonged and increased peripheral inputs. The use of lidocaine injections to control pain via regulating the peripheral pathways that play a role in the development of TTH supports this opinion.

Use of local anesthetics at the appropriate concentrations blocks nerve conduction. They exert their effects not only on nerve fibers (axons and dendrites), but also on the nerve body, myocardium, skeletal muscles, smooth muscles, and on other excitable cells by reversibly blocking the transmission of depolarization waves. Additionally, they temporarily block painful signal transmission to the central nervous system [18]. Depending on the concentration, local anesthetics decrease Na influx by blocking Na channels, and decrease the rate of depolarization in nerve fibers and other excitable cells. Finally, local anesthetics inhibit excitable cells’ ability to spontaneously discharge [19].

Recent research has highlighted the importance of the TPs in such painful conditions as primary headache disorders and myofascial pain syndrome. TPs might play a role in the perception of the severity of pain by sensitizing the neurons of the trigeminal caudal nucleus, which receive input from cephalic blood vessels and pericranial muscles [3,20]. It was reported that the primary mechanism in the development of TTH is sensitization of the central pathways due to prolonged nociceptive inputs that arise in the pericranial myofascial muscles [20,21]. High concentrations of bradykinin, CGRP, substance P, TNF-α, IL-1b, serotonin, and norepinephrine in the TPs located in the pericranial myofascial tissues have been shown histopathologically. These mediators are suggested to play a role in the pathogenesis of TTH [22]. Localization of the TPs vary in patients with primary headache disorders; however, the trapezius, posterior cervical paraspinal, sub-occipital, and sternocleidomastoid muscles are the most common muscle groups in which TPs are located [23]. Stretch, TENS, injection therapies , and dry needling have all shown benefit [24]. Saline injections are also known to be effective [25] and as we used saline at the placebo group, we also underline that lidocaine injections are more effective than saline injections.

The pain-relieving effect of local anesthetic injection into the TPs may be due to a mechanism involving a decrease in mediators that cause algesia, desensitization of free-nerve endings, inhibition of the spread of pain within the muscles, and then suppression of the perception of prolonged pain in peripheral tissues. Repeated injections might also increase this effect and provide long-lasting pain relief. In the present study we used 5 lidocaine injections in group 4 to increase its lidocaine’s efficacy by suppressing the perception of prolonged pain. Decreases in the frequency of pain and VAS scores recorded in group 4 posttreatment at the 2., 4. and 6. months showed that the treatment protocol was successful.

Venancio et al. [23] compared the efficacy of lidocaine, corticosteroid, and dry-needle injections into the myofascial TPs and reported that lidocaine was effective in the treatment of headache. In another study Venancio et al. [26] compared BoNTA, lidocaine, and dry-needle injection into the myofascial TPs and suggested that lidocaine is a cost-effective treatment option for headache. Calandre et al. [27] evaluated 12 patients with cluster headache (4 episodic and 8 chronic). They administered mepivacaine (3%) to 6 patients at the beginning of their attacks and 5 of the patients (85%) had no complaints within minutes. Prophylactic treatment was given to 7 patients, which prevented attacks in 6 of the patients (86%). Additionally, in the 8 patients with chronic cluster headache they observed that the frequency of attacks decreased more than 50%, and that the severity and duration of attacks decreased in 7 of the patients.

Giamberardino et al. showed the effects of local therapy of active myofascial trigger points on migraine symptoms in 87 migraine patients [28]. Tfelt-Hansen et al. injected 1.5% lidocaine and saline to the trigger points of 50 acute migraine patients. 28 patients gave response to therapy but no significant difference was present between two groups [29]. Garcia-Levia et al. [30] injected 10 mg of ropivacaine into the myofascial TPs in 52 patients with migraine. The severity of pain decreased by more than 50% in 9 patients and by 11%-49% in 19 patients. They reported that post-treatment 8 out of 30 patients with chronic migraine had episodic migraine. Martin Herrero et al. reported that referred pain induced by myofascial trigger points (MTP) and sleep disorders can be factors that contribute to chronic tension-type headache [31]. Alonso Blanco et al. reported that active trigger points (TrPs) in neck and shoulder muscles contribute to tension-type headache [32]. In the present study repeated lidocaine injections successfully controlled prolonged and increased peripheral pain inputs, and resulted in prolonged pain control by re-establishing the central response to the pain; these results are in agreement with those of other studies [22-28].

There may be shifting between the sub-groups of TTH. Patients with frequent ETTH are at risk of developing CTTH. The primary reason for shifting from ETTH to CTTH is analgesic overuse [3]. Patients that were young and female, and had no vocational education, familial disposition, a high work load, and frequent TTH at baseline had an increased risk of migraine [33]. As such, controlling ETTH before it transforms to CTTH or migraine is essential.

The main limitations of the present study are the small number of patients included and the short duration of follow-up; however, the data obtained have important implications for the treatment of frequent ETTH.

## Conclusions

As a result, post-treatment results of the lidocain injected groups were different from the pretreatment and the treatment found to be effective. According to the results, application of repeated lidocain injection is said to be an effective treatment approach that provides long-lasting state of well being. For putting out the efficacy of lidocain injections in frequent ETTH exactly, randomized, placebo-controlled and long term follow up studies with repetetive and with different doses of lidocain injections are needed.

## Competing interests

The authors declare that they have no competing interests.

## Authors’ contributions

KO has made the study design and injections were made by him. GHL has followed up and recorded the patients data, and wrote the manuscript. İLE has checked out the writing format and stastistical analysis. All authors read and approved the final manuscript.
